# Smartphone sensor-based depression detection in campus environments: a proof-of-concept study with small-sample behavioral analysis

**DOI:** 10.3389/fpsyt.2025.1468334

**Published:** 2025-08-07

**Authors:** Yichen Bai, Yueze Liu, Yang Zhang, Amr Tolba

**Affiliations:** ^1^ School of Information Science and Engineering, Lanzhou University, Lanzhou, China; ^2^ Cyberspace Administration of Lanzhou University, Lanzhou, China; ^3^ Computer Science Department, Community College, King Saud University, Riyadh, Saudi Arabia

**Keywords:** depression detection, feature engineering, daily mobile behavior analysis, smartphone sensors, machine learning, small data samples

## Abstract

**Introduction:**

Depression is a rising global health issue, particularly among adolescents, with university students facing distinct mental health challenges.

**Methods:**

This proof-of-concept study explores smartphone sensor-based depression detection in Chinese university campus settings using a small sample of 12 participants. We utilized data from accelerometers, gyroscopes, and light sensors to establish associations between smartphone-derived behavioral patterns and PHQ-9 scores, a standard depression measure. A customized data processing scheme tailored to campus life enabled the extraction of 18 feature sequences reflecting depressive symptoms. Feature selection was conducted using Pearson correlation, and model validation was performed using leave-one-out cross-validation with common classification algorithms.

**Results:**

The results yielded accuracy rates between 73.11% and 88.24%. Findings showed negative correlations between PHQ-9 scores and dietary regularity, bedtime, and physical activity levels.

**Discussion:**

This pioneering study highlights smartphone sensors' potential for early depression detection in Chinese higher education, supporting non-invasive mental health interventions.

## 1 Introduction

In recent years, particularly after the onset of the COVID-19 pandemic, depression has become increasingly prominent worldwide (1), with the number of patients showing a growing trend (2). According to the World Health Organization’s 2022 World Mental Health Report, based on 2019 data, approximately 970 million people globally were affected by mental health disorders, including about 14% of adolescents (aged 10–19, approximately 175 million), who also suffered from these disorders, accounting for roughly 18% of the total number of people with mental disorders, a proportion that may be higher than expected, highlighting the severity of mental health issues among adolescents (3). According to reports, 6.8% of adults in China experience depressive disorders over their lifetime, and 3.4% suffer from depression (4). Among the educated population, the proportion of depression problems is higher, especially prominent on university campuses (5). According to the “2022 National Mental Health Survey Report” by the Institute of Psychology, Chinese Academy of Sciences, in 2022, only half of college students (54.72%) were free from mental health risks (6). For college students, depression often manifests in both their academic and personal lives. Students struggling with depression tend to have poorer academic performance (7). In daily life, phenomena such as suicide, social withdrawal, and criminal behavior often co-occur with mental health issues (8, 9). In addition, depression can also pose risks to physical health, such as cardiovascular disease, immune system disorders, and digestive system diseases (10, 11).

Depression is increasingly common in our lives and can significantly affect how people experience their quality of life (12–14). Therefore, the importance of early identification, evaluation, and intervention of depression problems cannot be ignored (15–17). Early identification can help us detect mental health issues promptly, thus preventing further deterioration of the problem (18). Assessment, on the other hand, allows us to understand an individual’s mental health status, including their emotions, thoughts, and behavioral patterns (19–21). Intervention measures, such as psychotherapy (22), medication (23), social support (24), and adopting healthy lifestyles (25, 26), can effectively help improve mental health problems (27, 28). For example, for college students, the sources of pressure they face include academic, career planning, daily life, and romantic relationship issues (29). Through early identification and evaluation, we can quickly discover possible mental health problems that they may have. Then we can help them improve these problems by implementing scientific intervention strategies, such as providing psychological counseling (30), conducting mental health education activities, providing social support (31, 32), etc. Therefore, we should pay attention to and actively employ these strategies to improve mental health, improve quality of life, and advance the mental well-being of individuals and society as a whole.

Smartphones have emerged as powerful tools in mental health research (33, 34), leveraging their embedded sensors such as accelerometers, gyroscopes, and light detectors to passively collect behavioral data linked to psychological well-being. Prior studies have harnessed these capabilities to monitor daily patterns and infer mental states (35–41), with researchers like Insel suggesting that such ecological measurements can detect early indicators of mood disorders, including depression and mania (42). Forchuk and colleagues further demonstrated the feasibility of using mobile technology to track depressive symptoms, capturing real-time data on behavior and environmental context without disrupting users’ routines (43). However, existing mobilebased depression research has predominantly focused on general populations in non-academic settings. Although a limited number of studies have explored mental health in university contexts, substantial sociocultural variations across nations have led to a critical gap: the absence of investigations targeting the unique ecosystem of Chinese university campuses, where academic pressures, social dynamics, and cultural norms distinctively shape mental health trajectories. To address this gap, our study pioneers the investigation of depression correlates within Chinese higher education environments. We specifically aim to: (a) Establish multi-modal associations between smartphone-derived behavioral patterns (accelerometer/gyroscope/light sensor) and PHQ-9 scores, (b) Identify feature sequences reflecting depression symptomatology in academic settings, (c) Validate these digital biomarkers through comparative machine learning analysis (44, 45). Furthermore, we pioneered the investigation of campus-specific behavioral correlates of depression in Chinese higher education contexts. We summarize our main contributions as follows.

1. Leveraging smartphones as data collection terminals, we designed a sensor data processing and filtering scheme tailored to the campus life context of Chinese university students, and further developed several behavioral indicators of psychological traits based on this framework.
2. Building on the above, we employed various machine learning techniques to validate the effectiveness of these feature indicators, exploring the potential for AI-driven automated depression detection among university students.
3. We investigated the associative relationships between PHQ-9 scores and daily behavioral features of university students, offering a deeper analytical perspective on the psychological health traits underlying their routine activities.

The rest of the paper is organized as follows. Section 2 presents the research methods, including data collection, data preprocessing, feature extraction, feature selection, classification modeling, and model evaluation, along with detailed definitions of each feature. Section 3 provides the results derived from the features we extracted and the limitations of the current study and outlines future work.

## 2 Materials and methods

### 2.1 Data

From November 2023 to January 2024, we recruited 12 volunteers from Lanzhou University’s undergraduate and graduate students through online promotion to serve as our data collection subjects. We used PHQ-9 as a tool to obtain depression labels for each participant. Considering the subjective nature of PHQ-9 scores, we conducted a PHQ-9 questionnaire survey on volunteers every 3 days, removed volunteers with large differences in scale scores, and finally averaged them. Additionally, the experimental period was short enough that annual variations in sunlight exposure did not need to be considered.

#### 2.1.1 PHQ-9

The Patient Health Questionnaire-9 (PHQ-9) functions as a versatile tool, used to detect, diagnose, monitor and assess the severity of depression. It combines the Diagnostic and Statistical Manual of Mental Disorders, Fourth Edition (DSM-IV) diagnostic criteria for depression with additional major depressive symptoms, forming a brief self-reporting tool. The PHQ-9 consists of nine items, each corresponding to the DSM-IV diagnostic criteria for depression. The scores for each item range from 0 to 3, with a total score range of 0 to 27. Higher scores reflect greater severity of depression: scores below 5 suggest no depression, 5–9 suggest mild depression, 10–14 indicate moderate depression, 15–19 indicate moderately severe depression, and scores over 20 signify severe depression (46, 47).

#### 2.1.2 SensorData

SensorData is an application developed by us to collect smartphone sensor data. It can collect data from eight types of sensors: accelerometer, Bluetooth, gyroscope, light sensor, location information, orientation sensor, proximity sensor, and step counter. In this article, we only analyzed data from three smartphone sensors: accelerometer, gyroscope, and light sensor.

During the data collection process, each volunteer was assigned an anonymous account, such as “san1,” “san2,” “san3,” etc. The SensorData app collects only sensor data from volunteers and does not access any other information on their phones. This article primarily focused on studying and analyzing accelerometer, gyroscope, and light sensor data from smartphones. Therefore, Therefore, we set the SensorData recording frequency to 100*Hz* during data collection., as high-frequency data allows us to analyze volunteers’ smartphone usage more realistically and accurately.

#### 2.1.3 Data cleaning

**Figure 1:**
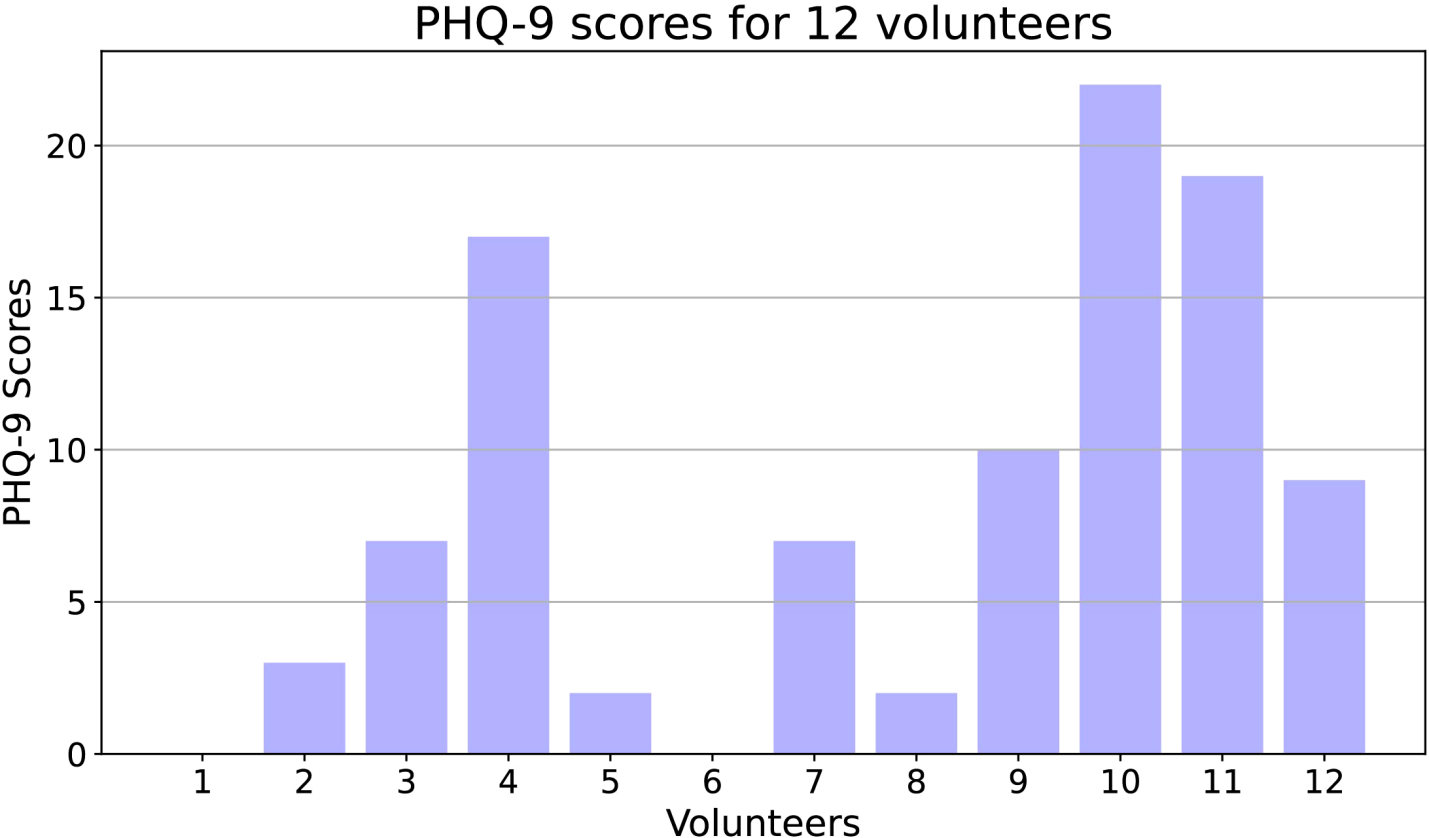
The PHQ-9 scores for 12 volunteers.

Data cleaning primarily targeted data with format errors and invalid entries. Specifically, we used regular expressions to match correctly formatted data and deleted incorrectly formatted data. Additionally, if a volunteer’s data for a particular day was insufficient for analysis, we removed that volunteer’s data for that day. In the end, we obtained data from a total of 132 days from 12 volunteers. There were 5 normal volunteers, 7 depressed volunteers, and 3 of them had PHQ-9 scores above 15. Specific PHQ-9 scores are shown in **Figure 1**.

### 2.2 Feature extraction

**Figure 2:**
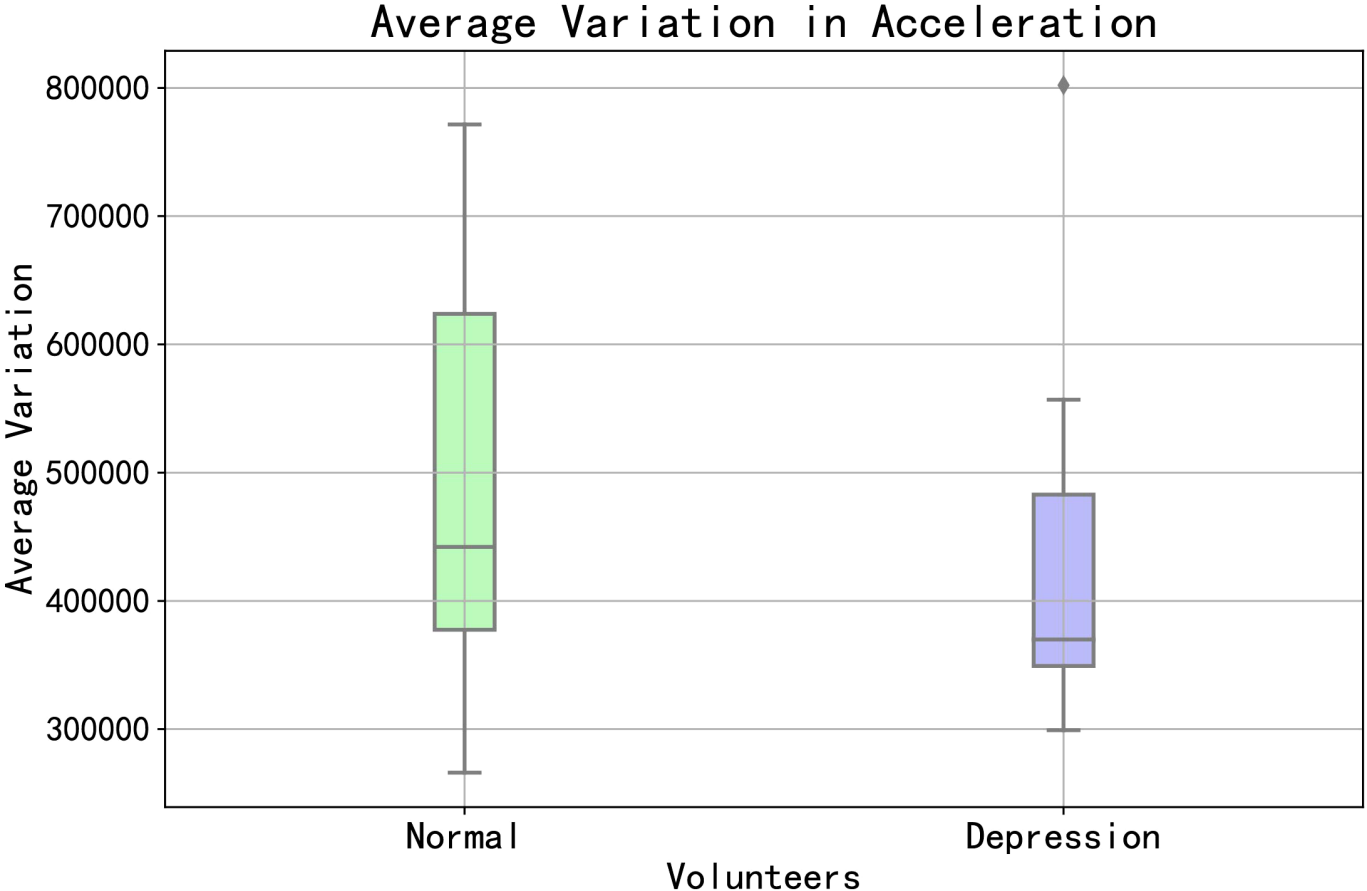
The total acceleration change between depression volunteers and normal volunteers.

The basis for feature extraction is the various types of mobile behaviors exhibited by volunteers during the data collection period. On the one hand, we can start by examining the overall situation of the volunteers, such as their overall movement patterns. As depicted in **Figure 2**, we categorized volunteers with PHQ-9 scores<5 as “Normal” and those with PHQ-9 scores ≥5 as “Depression”. We summed the accelerometer changes for both groups of volunteers to represent their overall movement patterns. From the figure, it can be observed that there are certain differences between normal and depressed volunteers.

**Figure 3:**
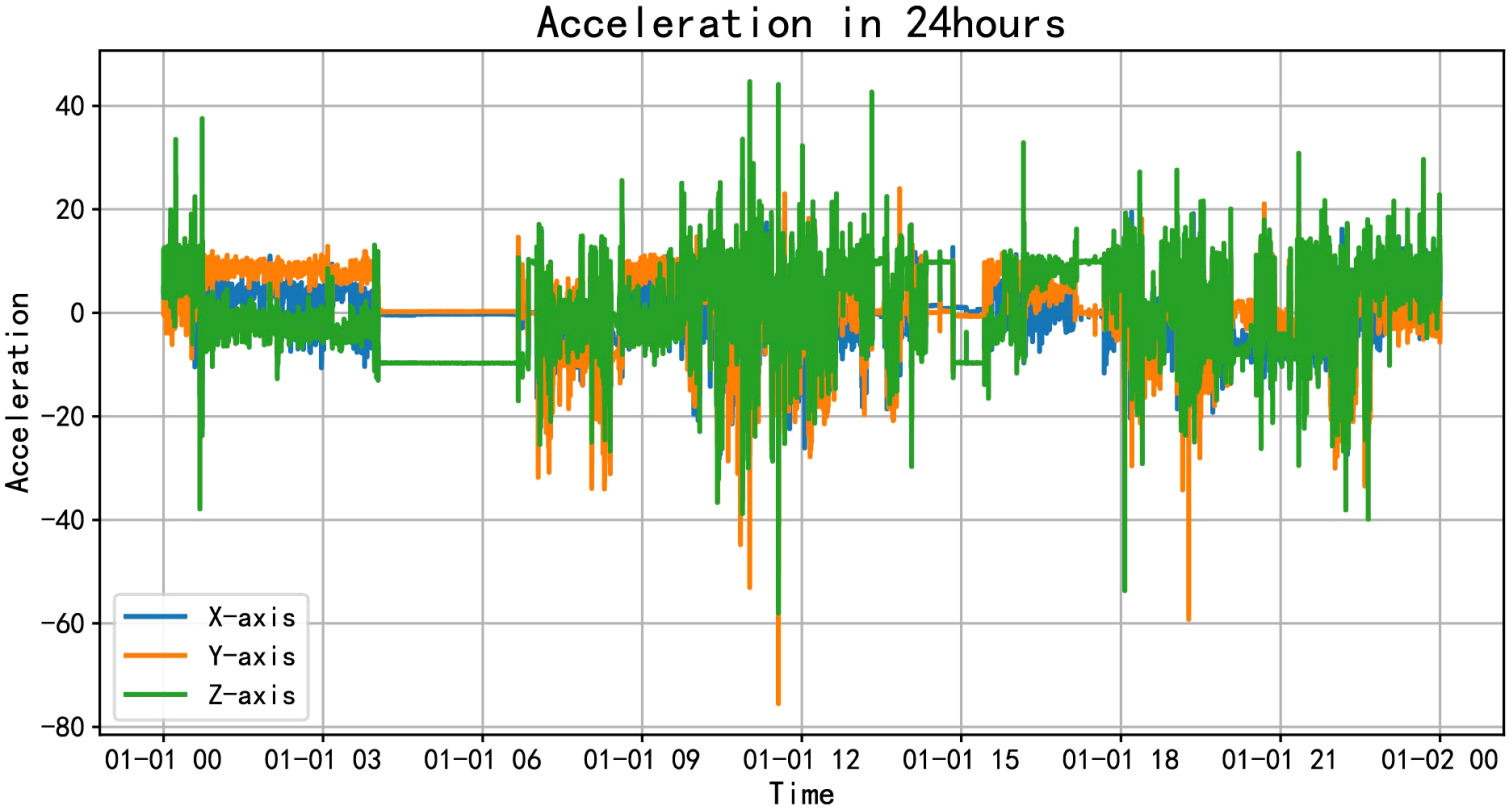
Accelerometer data throughout a day.

On the other hand, we can dive into the detailed activities of volunteers throughout the day, such as various activities they engage in. As shown in **Figure 3**, the accelerometer data throughout the day is visually presented. From the figure, it can be seen that volunteers start their activities around 8 o’clock, with more intense activity between 10 am and 12 pm, followed by a period of rest from 12:30 pm to 1:30 pm, and then activities continue from 2 pm to 9 pm, gradually decreasing in intensity afterward. By combining the data characteristics with their corresponding times, we infer various mobile behaviors of the volunteers: waking up, attending classes, having lunch, taking a nap, having dinner and going to sleep, among others. We can extract features from aspects such as the timing and frequency of these activities.

In this article, by analyzing accelerometer data, gyroscope data, and light sensor data, we extracted 37 possible features.

### 2.3 Feature selection

We used Pearson correlation coefficient to filter the features. The calculation formula is as shown in Equation 1.


(1) $$\rho_{X,Y}=\frac{\sum (X_{i}-\bar{X})\left(Y_{i}-\bar{Y}\right)}{\sqrt{\sum {\left(X_{i}-\bar{X}\right)}^{2}\cdot \sum {\left(Y_{i}-\bar{Y}\right)}^{2}}}$$

where:

• *X_i_* and *Y_i_* are the feature values and label values of the sample points.

• $\bar{X}$ and $\bar{Y}$ are the means of the feature values and label values, respectively.

Regarding the correlation between features and the severity of depression, we retained features with higher correlation; for the correlation between features, we retained features with lower correlation. Specifically, regarding the correlation between features and the severity of depression, we set the threshold to 0.2. Features with a correlation coefficient ≥0.2 were retained, otherwise they were deleted. Regarding the correlation between features, we set the threshold to 0.8. Feature pairs with a correlation coefficient ≥0.8 had one feature retained, and the other feature deleted (48). In the end, through this filtering process, we obtained 18 features. The feature sequence is as follows:

#### 2.3.1 Acceleration absolute change rate

Considering that the variations in acceleration data can be either positive or negative, the absolute value of these changes effectively reflects the overall magnitude of acceleration alterations. The acceleration rate of change is calculated over a specific time interval by averaging the absolute differences between consecutive acceleration data points. Specifically, we subtract the acceleration data at adjacent time points to obtain the change, then compute the average of all these changes, sum them up, and divide by the total number of data points. This process yields the average rate of acceleration change during that time period. The calculation formula is as shown in Equation 2.


(2) $$\text{AARC}=\frac{\sum_{t=1}^{n-1}\left|a_{t+1}-a_{t}\right|}{n-1}$$

where:

• *n* is the total number of collected acceleration data points;

• *a_t_* represents the acceleration value at time *t*;

• $\left|a_{t+1}-a_{t}\right|$ denotes the absolute difference between consecutive acceleration values;

• $\sum_{t=1}^{n-1}\left|a_{t+1}-a_{t}\right|$ sums up the absolute differences between consecutive acceleration values.

From this feature, we can infer how much and how little activity a volunteer has in a day.

#### 2.3.2 Average acceleration

Calculate the average of the volunteer’s accelerometer sensor data.This feature reflects the total amount of exercise a volunteer gets in a day.

#### 2.3.3 Gyroscope absolute change rate

When we turn the phone, the value of the gyroscope changes.Similar to the acceleration data, we consider that the change in the gyroscope data may be positive or negative, and the absolute value of the change can well reflect the total change amplitude of the gyroscope data. The gyroscope absolute change rate is the average absolute change in gyroscope data continuously collected over a certain period of time. Specifically, subtract the gyroscope data between two adjacent time points to get the change, then take the absolute value of all changes, sum them, and divide by the total number of data points to get the absolute average rate of gyroscope change during this time period. The GACR can reflect the change of the status of the mobile phone, which indirectly reflects the movement of the volunteers.

#### 2.3.4 Frequency of gyroscope absolute value greater than 0.1

By analyzing the data, we found that when the volunteers exercised more vigorously, the gyroscope sensor data would be above 0.1. Count the number of times the absolute value of the gyroscope data is greater than 0.1, and divide it by the total amount of data to get the frequency of the absolute value greater than 0.1 in the data. This feature can reflect the frequency of volunteer exercise.

#### 2.3.5 Average gyroscope

Calculate the average of the volunteer’s gyroscope sensor data.

#### 2.3.6 Average light

The average light is the sum of the light sensor data continuously collected over a certain period of time divided by the total number of data points. Specifically, sum all the light sensor data, then divide the sum by the total amount of data to get the average value of the light sensor data during this time period. The “Average Light” feature directly reflects the brightness of the volunteers’ environment. In addition, we analyzed the data of the depressed volunteers and the data of the normal volunteers, and found that the more time the volunteer spent outside, the higher his average light.

#### 2.3.7 Frequency of light intensity values greater than 1000

After analysis, we found that in the light sensor data, “1000” is a key value to distinguish between volunteers outdoors or indoors. Whenever the volunteers went out during the day, the light sensor value was generally above 1000, and when indoors, the sensor value was generally below 1000. We count the number of occurrences of light sensor data greater than 1000, and divide by the total amount of data to obtain the occurrence frequency of data greater than 1000. From this feature, we can predict the proportion of time volunteers spend outside during the day.

#### 2.3.8 IQR

We use the interquartile range to analyze the degree of variation in the dataset. This metric captures the middle 50% range of the data, specifically the interval from the first quartile (Q1) to the third quartile (Q3). Given a dataset {*x*_1_ *,x*_2_ *,…,x_n_*}, after sorting, it becomes {*x*_(1)_ *,x*_(2)_ *,…,x*_(_ *_n_*_)_}, and:

- The first quartile *Q*1 is the value at the (*n* +1)*/*4 position in the sorted dataset (if this position is not an integer, the mean of the two adjacent numbers is taken).
- The third quartile *Q*3 is the value at the 3(*n* +1)*/*4 position in the sorted dataset (if this position is not an integer, the mean of the two adjacent numbers is taken).
- The formula for IQR is as shown in Equation 3.

(3) IQR=Q3−Q1

Using accelerometer data as an example, we can understand the volatility of the data and analyze movement patterns and behavioral characteristics through the interquartile range in the X-axis, Y-axis, and Z-axis directions.

#### 2.3.9 Skewness

We use skewness as a time-domain feature to analyze the asymmetry and skewness of data distribution, determine the shape of the data distribution, and extract signal characteristics of different movement patterns. Given a dataset {*x*_1_ *,x*_2_ *,…,x_n_*}, with a mean of $\bar{x}$ , and a standard deviation of *σ*, the formula for skewness is shown as Equation 4.


(4) $$\text{Skewness}=\frac{n}{\left(n-1\right)\left(n-2\right)}\sum_{i-1}^{n}{\left(\frac{x_{i}-\bar{x}}{\sigma }\right)}^{3}$$

where:

- *n* is the number of data points,
-
$\bar{x}$ is the mean of the data,
- *σ* is the standard deviation of the data,
- *x_i_* is the i-th data point.

#### 2.3.10 Spectral bandwidth

We use spectral bandwidth as a frequency-domain feature to analyze the concentration of the signal spectrum. It is obtained by calculating the power spectral density (PSD) and is described using the weighted average frequency and the second moment. The specific formula is as follows: Given the power spectral density of the signal as *P*(*f*), and the frequency as *f*, then the relevant formulas are shown in Equations 5 and 6:


(5) $$f_{c}=\frac{\sum_{i}f_{i}P\left(f_{i}\right)}{\sum_{i}P\left(f_{i}\right)}$$

(6) $$\text{Bandwidth}=\sqrt{\frac{\sum_{i}{\left(f_{i}-f_{c}\right)}^{2}P\left(f_{i}\right)}{\sum_{i}P\left(f_{i}\right)}}$$

where:

- *P*(*f_i_*) is the power spectral density corresponding to the i-th frequency component,
- *f_i_* is the i-th frequency component,
- *f_c_* is the weighted average frequency.

#### 2.3.11 Spectral slope

We use spectral slope as a frequency-domain feature to describe the trend of the signal power density with frequency. It can be calculated by performing a linear regression on the power spectral density (PSD) of the signal. The specific formula is as follows: Given *f* as the frequency and *P*(*f*) as the power spectral density at frequency *f*, the formula for calculating SS is as shown in Equation 7.


(7) $$SS=\frac{N\sum_{i=1}^{N}f_{i}P\left(f_{i}\right)-\sum_{i=1}^{N}f_{i}\sum_{i=1}^{N}P\left(f_{i}\right)}{N\sum_{i=1}^{N}f_{i}^{2}-{\left(\sum_{i=1}^{N}f_{i}\right)}^{2}}$$

where:

- *N* is the number of frequency components,
- *f_i_* is the i-th frequency component,
- *P*(*f_i_*) is the power spectral density corresponding to the i-th frequency component.

Using accelerometer data as an example, we can infer the intensity of the volunteers’ movements through the spectral slope over different time periods.

#### 2.3.12 Acceleration-based bedtime prediction

When we hold our mobile phones, there is inevitably some movement or changes in posture, which leads to continuous changes in the phone’s acceleration data. When we put down the phone to prepare for sleep, the phone remains stationary. The first time to meet the condition is the bedtime. Therefore, we can infer the volunteer’s bedtime based on the changes in the phone’s acceleration. Acceleration-Based Bedtime prediction involves determining whether the change in acceleration data continuously collected over a certain period of time after the current moment (e.g., the subsequent 5000 data points) falls within a specific range (± 2), in order to predict the volunteer’s bedtime. Specifically, if within the subsequent 5000 data points, the acceleration change between each data point remains within the ±2 range, and the current moment *t* is between midnight (0:00) and 5:00 AM, then the data corresponding to the current moment is marked as the bedtime.

#### 2.3.13 Acceleration wake-up prediction

Through communication with volunteers, we found that most volunteers have a habit of checking phone messages after waking up. Therefore, we can infer the volunteer’s wake-up time by analyzing their morning acceleration data. Acceleration wake-up prediction involves predicting the volunteer’s wake-up time by judging whether the change in acceleration data continuously collected over a certain period of time after the current moment falls within a specific range. Specifically, if within the subsequent 3000 data points, 50% of the data have an absolute acceleration change greater than or equal to 2, then the data corresponding to the current moment is marked as the wake-up time.

#### 2.3.14 Morning out

Morning outings refer to a specific time period during which we check whether the light sensor data shows a light value greater than or equal to 1000 in the morning (from 7 a.m. to 10 a.m.). Additionally, within the subsequent 5000 accelerometer data points, if 70% of the data have an absolute acceleration change greater than 3, we infer that the volunteer went out in the morning (e.g., for morning exercises, breakfast, or classes).

#### 2.3.15 Noon out

Similar to morning outings, noon outings involve checking whether the light sensor data shows a light value greater than or equal to 1000 during noon hours (from 11 a.m. to 1 p.m.). Additionally, within the subsequent 5000 accelerometer data points, if 70% of the data have an absolute acceleration change greater than 3, we infer that the volunteer went out at noon (e.g., for lunch).

#### 2.3.16 Afternoon out

Similar to morning and noon outings, afternoon outings involve checking whether the light sensor data shows a light value greater than or equal to 1000 during the afternoon (from 2 p.m. to 5 p.m.). Additionally, within the subsequent 5000 accelerometer data points, if 70% of the data have an absolute acceleration change greater than 3, we infer that the volunteer went out in the afternoon (e.g., for exercise or classes).

#### 2.3.17 Evening out

During the evening, when light intensity decreases, we found that there is little difference between indoor and outdoor environments due to artificial lighting. Therefore, we cannot use light sensor data for analysis during this time. Instead, we analyze accelerometer sensor data. Evening outings involve checking whether the accelerometer data shows an absolute acceleration change greater than 3 within the subsequent 5000 accelerometer data points during the evening (from 5 p.m. to 7 p.m.). We infer that the volunteer went out in the evening (e.g., for dinner).

#### 2.3.18 Light-based bedtime prediction

By analyzing light data, we observed that when volunteers turn off their phone screens at night, the light sensor values fall within the range of 0 to 1. We can utilize this range to determine when the volunteer puts down the phone and begins to prepare for sleep. Light-based bedtime prediction involves predicting the volunteer’s bedtime preparation time by judging whether the subsequent continuous 1000 light sensor data points all fall within a specific range. Specifically, if each data point’s light value remains within 1, then the data corresponding to the current moment is marked as the sleep time point.

We computed the Pearson correlation coefficient for the above 18 characteristics and the degree of depression. If the result is an integer, it means that the two are positively correlated, and if it is not, it is negative. Among all the features, the correlation coefficient between feature AB and depression degree is the highest, and the correlation coefficient between feature Skewness and SS and depression degree is the lowest. The detailed results are shown in **Supplementary Table S1** in the **Supplementary Material**.

### 2.4 Classification modeling

#### 2.4.1 Dataset division

Due to the small scale of the dataset, we used the Leave-One-Out method for dataset division. LeaveOne-Out Cross Validation (LOO-CV) is a special type of cross-validation method (49). Specifically, suppose we have a dataset *D*, which contains *N* samples. For each iteration *i* (*i* = 1,2*,…,N*), we use all data except the *i*-th sample as the training set, denoted as *D*_train_ *_i_*, and use the *i*-th sample as the test set, denoted as *D*_test_ *_i_*. We train the model with the training set, and then calculate the error *E_i_* of the model with the test set. FFinally, we calculate the average error *E* of *N* iterations, which is the final generalization error of the model. The formula for calculating *E* is as shown in Equation 8.


(8) $$E=\frac{1}{N}\sum_{i=1}^{n}E_{i}$$

where, *E_i_* is the error of the i-th iteration.

In this article, the dataset contains samples from 12 individuals over 132 days. Each time, we take out one sample from the dataset as the test set, and the remaining 131 samples serve as the training set. In this way, each sample in the dataset has one opportunity to be used as the test set alone, while the remaining samples are used to train the model (50).

#### 2.4.2 Model construction and evaluation

We considered using machine learning to construct the model (51, 52). When marking labels, to enhance the model’s accuracy, we marked the volunteer data with PHQ-9 scores< 5 as 0, representing asymptomatic data; and marked the volunteer data with PHQ-9 scores ≥5 as 1, representing depressive symptom data. In this article, we used five machine learning algorithms, Support Vector Machine (SVM), Decision Tree (DT), K-Nearest Neighbor (KNN), Naive Bayes (NB), and Random Forest (RF), to construct the model (53). In our article, we used the Leave-One-Out method to divide the dataset. Therefore, we do not have a fixed test set, nor do we need to specifically divide a test set for verification. This allows us to directly evaluate and compare the performance of different machine learning models by comparing information such as accuracy, recall, precision, and F1 scores of different models.

### 2.5 Evaluation results

We calculated the accuracy, recall, precision, and F1 scores for four machine learning models: SVM, DT, KNN, NB, and RF. Our calculation formula utilizes four key indicators from the confusion matrix, namely True Positive (TP), False Positive (FP), True Negative (TN), and False Negative (FN) (54). Here, we used “Accuracy” as the final indicator to evaluate each model. By comparison, we found that: RF *>* KNN *>* DT *>* SVM *>* NB, as shown in **Table 1**.

**Table 1:** Evaluation results for five models.

Model	Accuracy	Precision	Recall	F1 Score
SVM	73.94%	86.55%	87.39%	73.95%
DT	74.79%	84.87%	89.92%	74.79%
KNN	78.99%	89.92%	89.08%	78.99%
NB	73.11%	76.47%	96.64%	73.11%
RF	88.24%	94.96%	93.28%	88.24%

## 3 Discussion

### 3.1 Results

**Figure 4:**
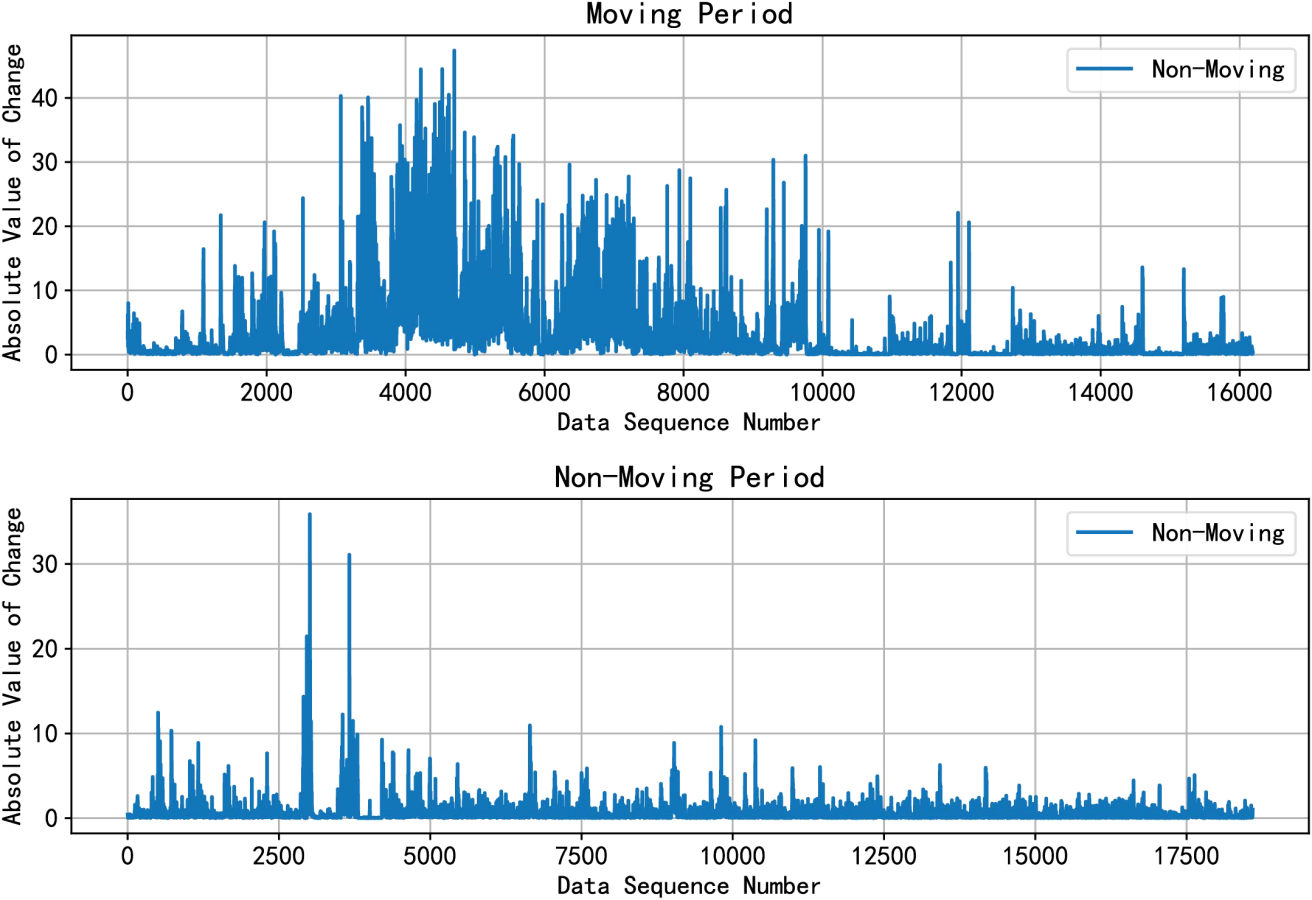
Acceleration change degree between moving period and non-moving period.

In this article, we extracted 18 feature sequences that effectively reflect the connection between mobile phone usage patterns and depression symptoms. We also verified the association between mobile phone sensor data and depressive symptoms. During the feature extraction phase, in addition to basic features such as mean and rate of change, we based our analysis on daily life behaviors to extract features like wake-up time, bedtime, and frequency of outdoor activities. We found that the volunteer’s mobile phone sensor data could represent real-time movement behaviors in the form of these features, as shown in **Figure 4**. For instance: When volunteers are running, the accelerometer and gyroscope sensor data exhibit intense changes lasting more than 10 minutes. During walking, the accelerometer and gyroscope data changes are less pronounced compared to running. In a stationary state, the sensor data changes show smaller variations.

**Figure 5:**
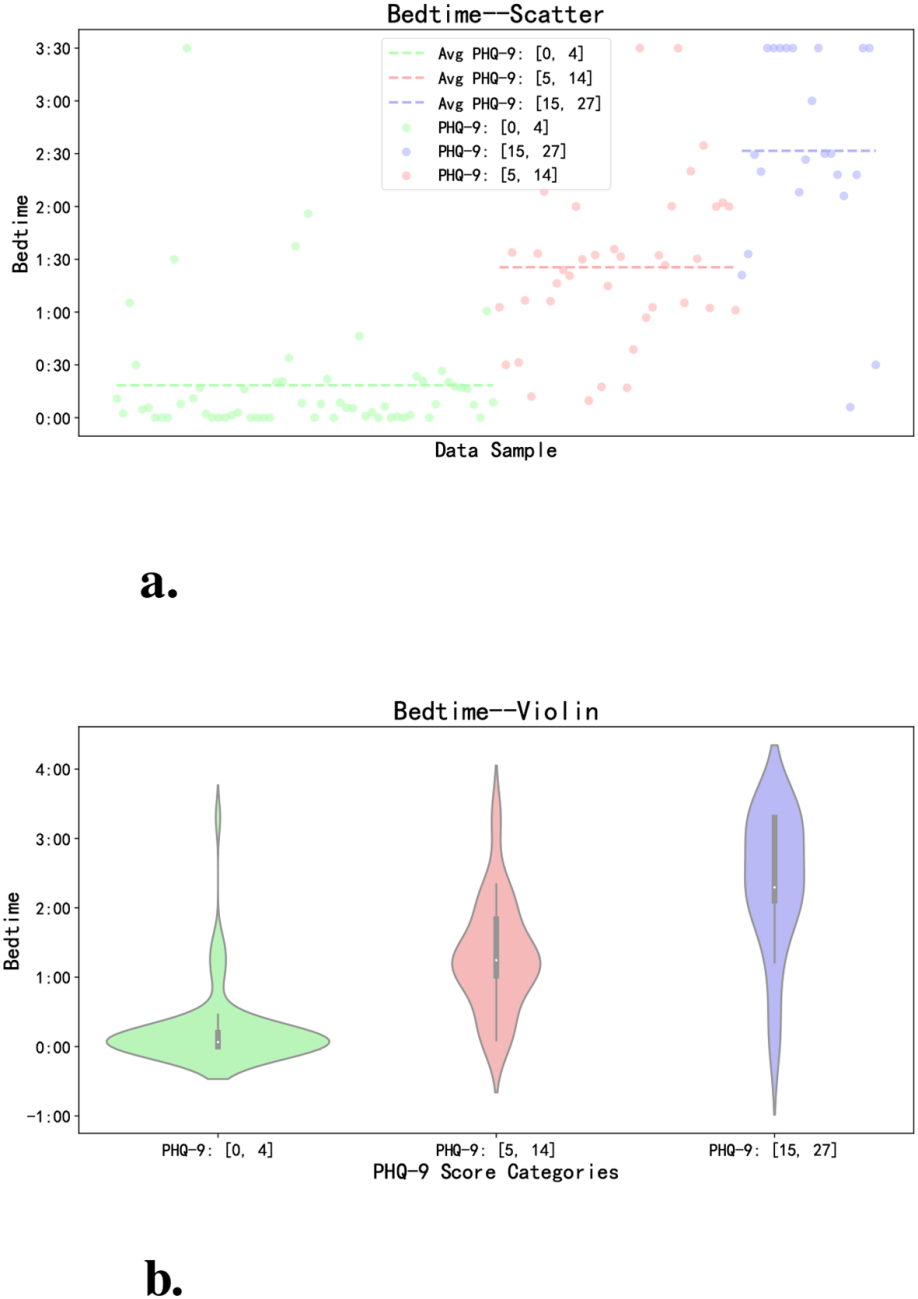
Comparison of bedtime of volunteers with different PHQ-9 scores. **(a)** The scatter plot intuitively reflects the distribution of different volunteers’ sleeping time every day. **(b)** The violin plot directly reflects the distribution of sleep time of different categories of volunteers.

Furthermore, this article revealed a correlation between most volunteers’ PHQ-9 scores and their feature values. For example: In terms of getting to sleep at night, although volunteers exhibit different sleep habits, we can identify certain patterns among different groups based on their PHQ-9 scores. We take the feature ‘Acceleration-Based Bedtime Prediction’ as an example to illustrate the sleep characteristics of volunteers in different PHQ-9 categories. As shown in **Figure 5a**, volunteers with PHQ-9 scores between [0, 4] have more concentrated points, indicating a more regular bedtime, usually between 0:00 and 0:30, with an average bedtime of 0:21. However, for volunteers with scores between [5, 14] and [15, 27], their points are more dispersed, representing irregular bedtimes, ranging from 0:15 to 3:30. Moreover, as the scores increase, the average bedtime becomes later. In **Figure 5b**, we can more intuitively observe that as the PHQ-9 scores rise, the shapes change from flat to elongated, indicating an increasing irregularity in bedtimes.

**Figure 6:**
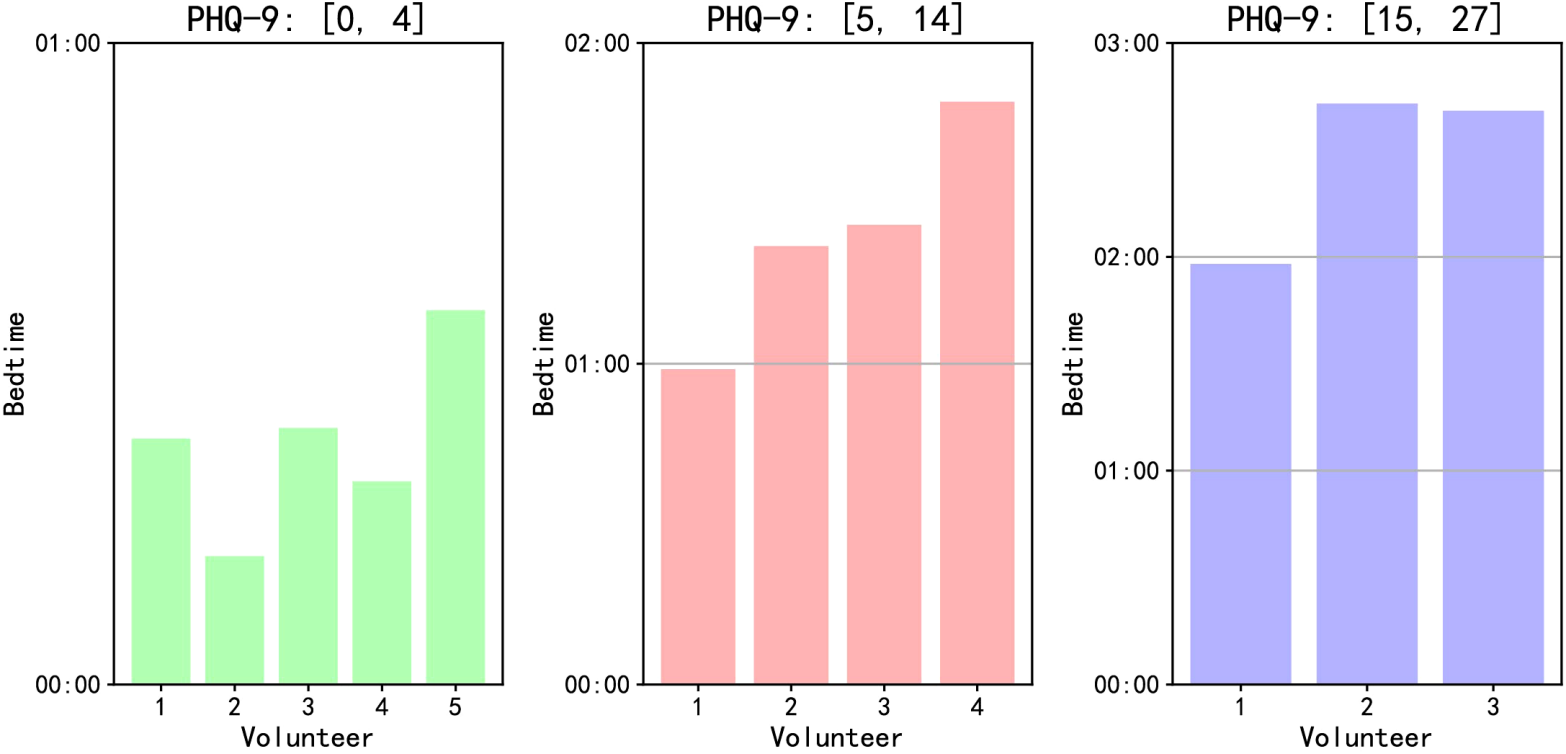
Average bedtime of volunteers with different PHQ-9 scores.

On the overall level, we analyze based on each volunteer’s average bedtime. As illustrated in **Figure 6**, volunteers with PHQ-9 scores between [0, 4] tend to fall asleep earlier on average, those with scores between [5, 14] tend to fall asleep later, and those with scores [15, 27] have the latest average bedtime. Through face-to-face interactions with volunteers, we have observed that some individuals with higher PHQ-9 scores have developed the habit of staying up late using their smartphones for activities such as watching videos or reading novels, consequently experiencing frequent episodes of insomnia. Conversely, volunteers with lower PHQ-9 scores typically find it easier to fall asleep, with insomnia occurring less frequently.

**Figure 7:**
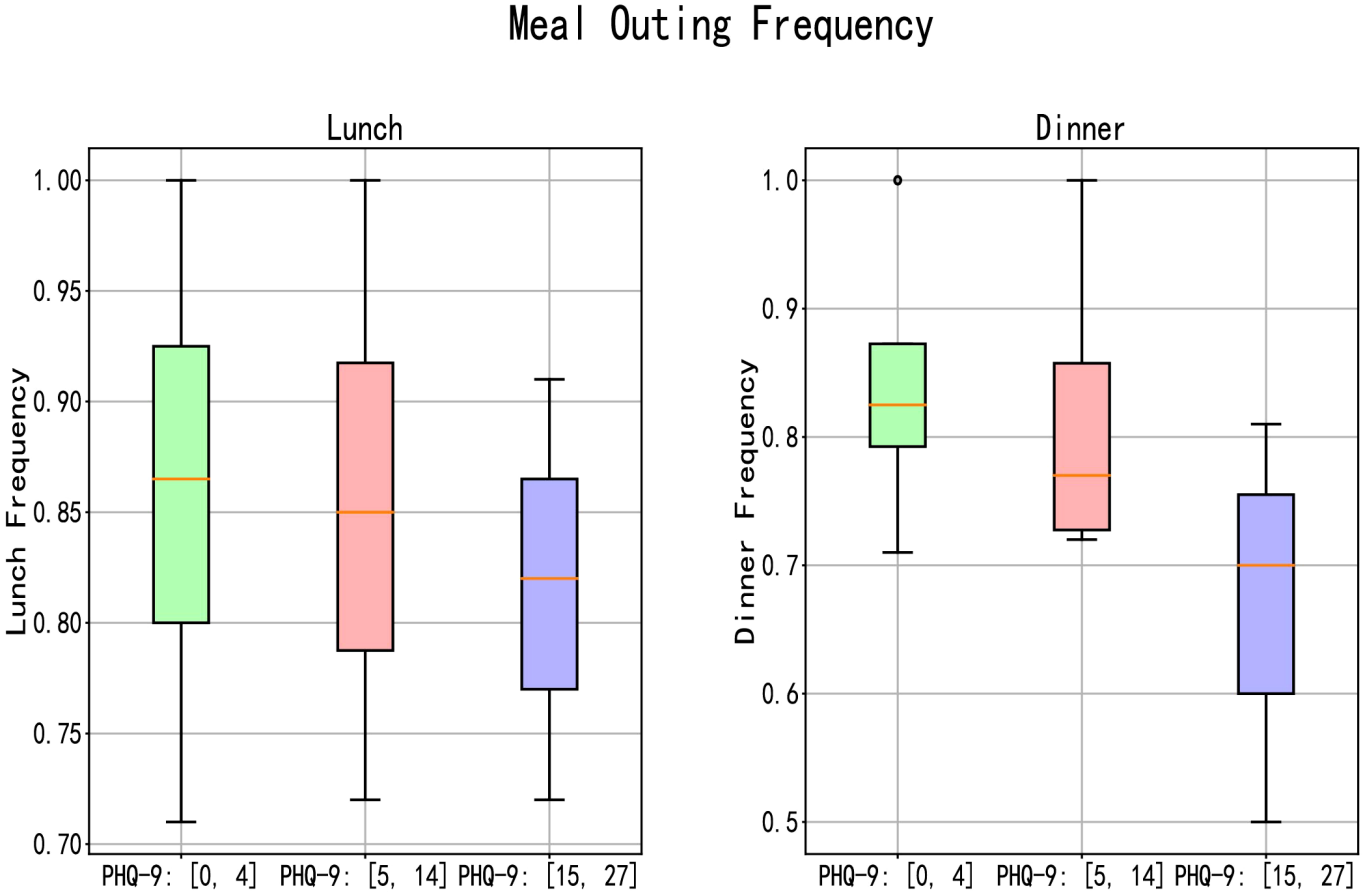
The frequency of regular lunch and dinner outings among volunteers with different PHQ-9 scores.

In terms of outing frequency, volunteers with different PHQ-9 scores also demonstrate variations in behavior. We use the features of “Noon Out” and “Evening Out” as examples to elaborate on this point. According to our survey conducted at Lanzhou University, students typically have lunch and dinner in the cafeteria on campus, with a small fraction opting for food delivery services, which can be delivered directly to dormitories or academic buildings. By comparing these two features, we observe that volunteers with PHQ-9 scores between [0, 4] have a higher frequency of timely outings for lunch and dinner, while those with scores between [5, 14] exhibit slightly lower rates of timely lunch outings, and volunteers with scores between [15, 27] have the lowest frequency, as depicted in **Figure 7**. Furthermore, while there is not much difference in lunch frequency among the three groups of volunteers, disparities emerge primarily during dinner.

**Figure 8:**
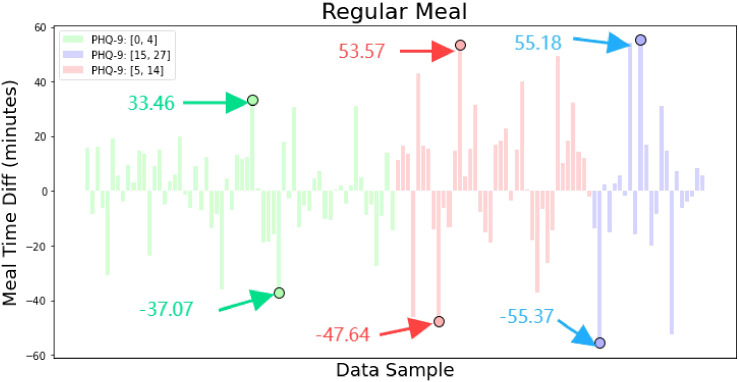
Difference between each volunteer’s daily meal time and their average meal time, used to analyze the regularity of eating habits among different volunteers. The graph shows the meal time deviation for each volunteer over a period of days, highlighting patterns of regularity or irregularity in their eating habits.

We subtracted each volunteer’s daily meal time from their average meal time to analyze the regularity of different volunteers’ eating habits based on this difference. As shown in **Figure 8**, the difference for most people is within ± 20 minutes, but volunteers with PHQ-9 scores between [0, 4] have a smaller average difference. As the score increases, the average height of the bars in the histogram also increases. The marked points in the figure represent the extreme values of the differences for different categories of volunteers. We can see that volunteers with scores between [15, 27] have the largest absolute extreme values, followed by those with scores between [5, 14], and the normal volunteers have the smallest. This reflects the characteristics of different categories of volunteers in terms of eating regularity. Through communication with volunteers, we found that some individuals with higher PHQ-9 scores prefer ordering takeout for dinner due to its convenience, while others with higher scores have irregular meal times, often eating too early or too late.

**Figure 9:**
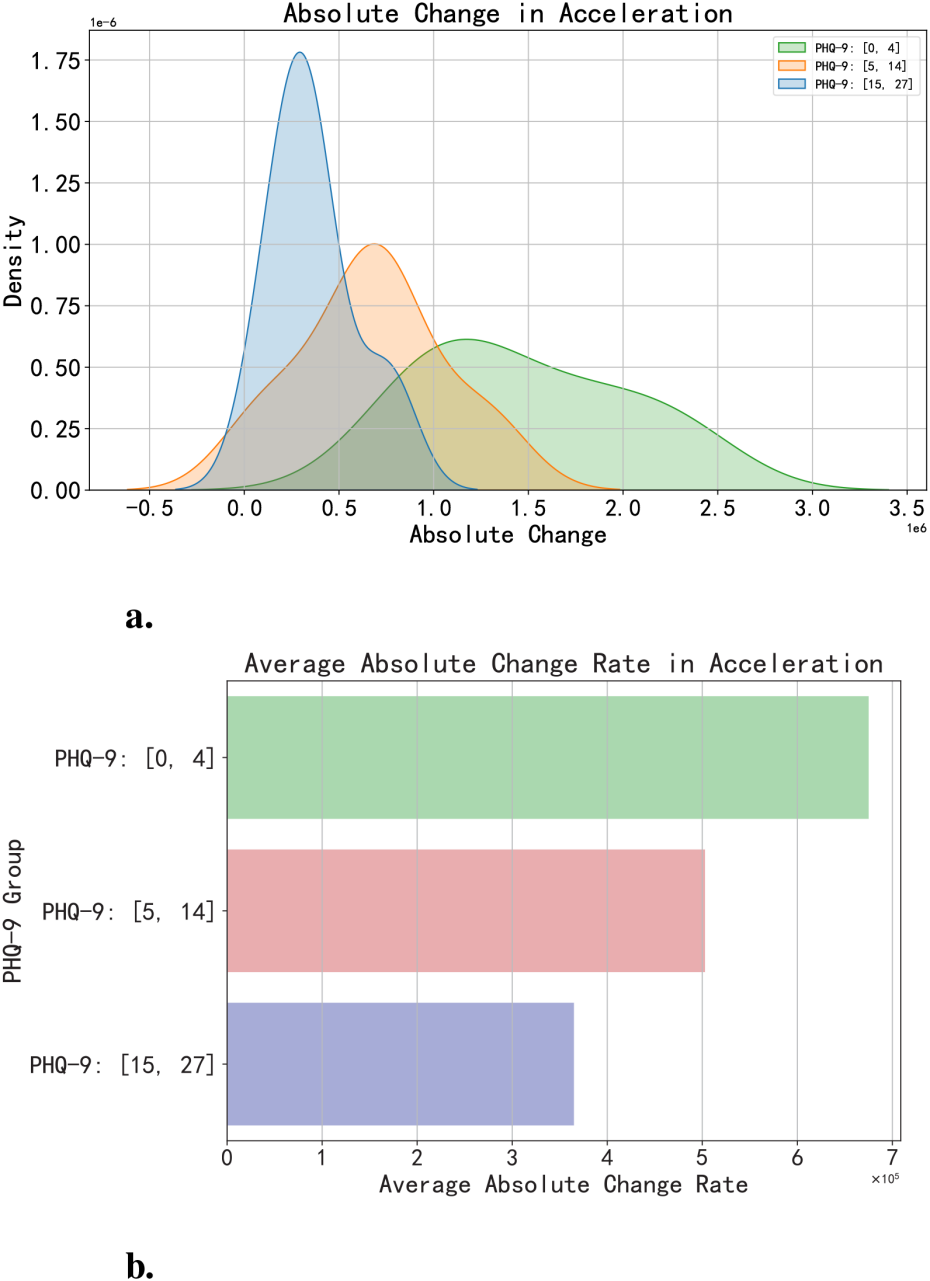
Comparison of daily physical activity of volunteers with different PHQ-9 scores. **(a)** This represents the absolute change in accelerometer data. **(b)** This represents the absolute change rate in accelerometer data.

In terms of daily physical activity, volunteers with different PHQ-9 scores also exhibit some distinctions. In this article, we utilized the absolute change rates of accelerometer and gyroscope data as indicators of volunteers’ physical activity levels. Taking the absolute change in accelerometer data as an example, as shown in **Figure 9a**, by comparing the physical activity levels of volunteers categorized into three groups based on PHQ-9 scores (0-4, 5-14, and 15 and above), we can discern differences from the peak abscissa values of the three sets of figures. Volunteers with PHQ-9 scores between [0, 4] demonstrate the highest peak abscissa values of absolute change in accelerometer data, followed by those with scores between [5, 14], and finally by volunteers with scores [15, 27]. **Figure 9b** displays the absolute change rates of accelerometer data for the three groups of volunteers. It reflects that, among the volunteers in this research, those with higher PHQ-9 scores tend to have relatively lower average levels of physical activity.

**Figure 10:**
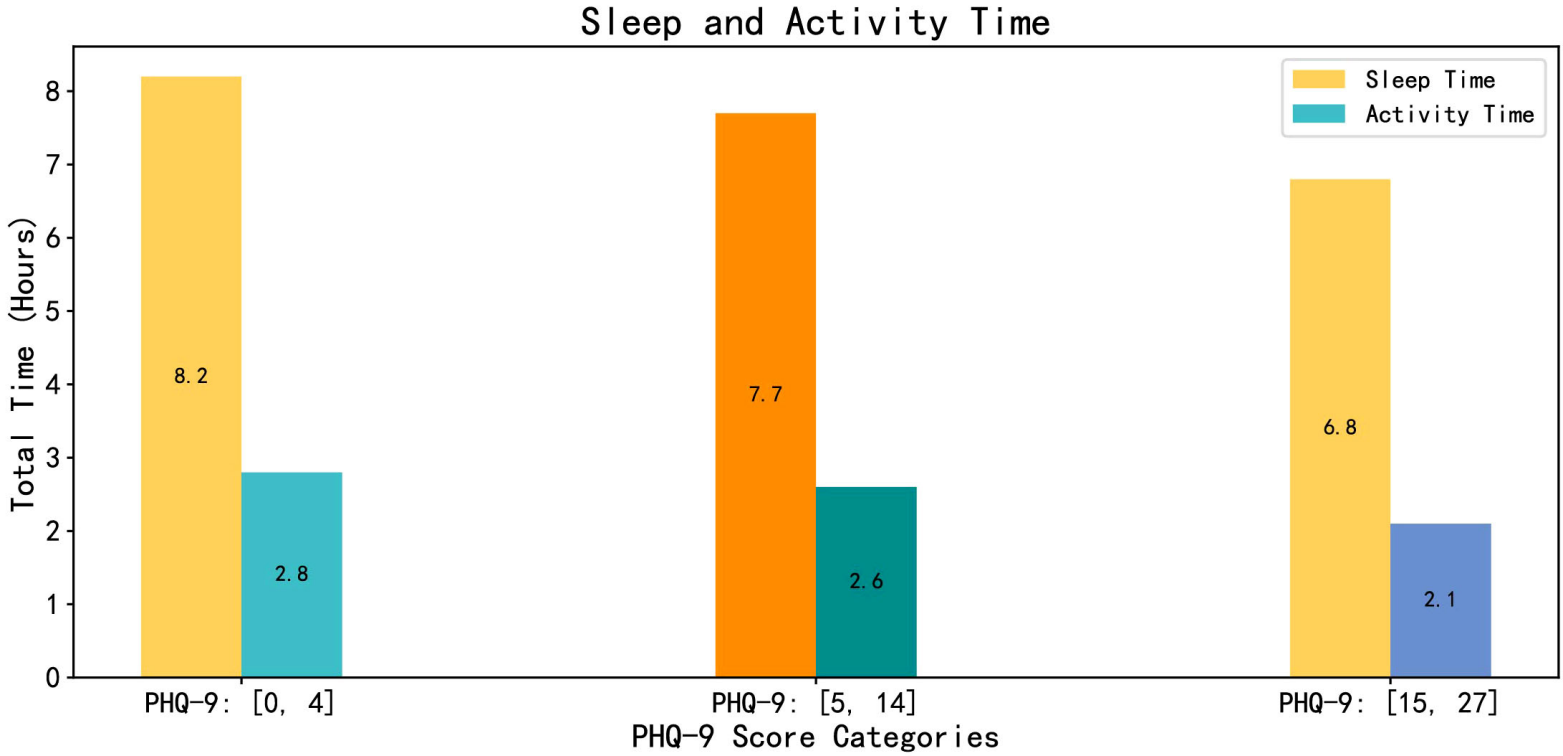
The total sleep time and activity time in volunteers with different PHQ-9 scores.

We integrated the volunteers’ sleep time with their activity time. Sleep time was determined using the features ‘Acceleration Wake-Up Prediction’ and ‘Acceleration-Based Bedtime Prediction.’ For activity time, we calculated the total duration for which the absolute value of the difference between successive acceleration sensor data points exceeded 2.5, representing the volunteers’ activity time. As shown in **Figure 10**, volunteers with PHQ-9 scores between [0, 4] exhibited the longest sleep and activity times. As the scores increased, both sleep and activity times decreased. Through face-to-face conversations with different volunteers, we discovered that those with higher PHQ-9 scores experienced difficulties falling asleep, frequent awakenings, and often spent time alone, feeling unmotivated to exercise. This article findings align with the actual characteristics observed in the volunteers.

However, it is important to note that these findings are currently specific to the Lanzhou University campus we studied.

### 3.2 Limitation

First, in the article, we marked the subjects’ labels based on the results of the PHQ-9 scale, which has certain limitations. Although PHQ-9 is a very effective depression measurement method, the results of the scale filling will inevitably be affected by other factors of the volunteers, leading to errors in the results, such as the possibility of volunteers filling in the questionnaire falsely, volunteers have a bias in understanding themselves, etc (55).

Second, the dataset is small. The number of volunteers we recruited is small, and some volunteers’ data is not available, resulting in a smaller final dataset size. This limited sample size inherently constrains the generalizability of machine learning models, potentially leading to overestimated classification accuracy due to increased variance and reduced model stability (56). Specifically, small-sample effects may amplify spurious correlations between sensor features and PHQ-9 scores, compromising the extraction of universal depression biomarkers. The current dataset may not necessarily represent the typical characteristics of depression patients. Subsequent research can expand the dataset size and recruit more representative volunteers (57).

Third, in this article, the type of data we analyzed is relatively limited. We use acceleration and gyroscope data as the volunteer’s movement data, but this cannot accurately represent the volunteer’s movement situation. Sports bracelets can better reflect the movement situation, such as steps, heart rate, etc. In addition, in this research, we can only determine the time when the volunteer puts down the phone and starts to sleep, but we cannot determine the real sleep time of the volunteer. This problem can also be solved by bracelet data.

Fourth, identification and evaluation of depression symptoms must go through complex medical and psychological procedures, the results of our evaluation method cannot replace the results of medical evaluation (37). We use this lightweight, non-disturbing data collection and analysis to carry out preliminary identification of depression symptoms. Users can refer to the preliminary identification results of depression symptoms, go to hospitals and other institutions, and carry out subsequent systematic inspections.

Finally, the research protocol was not preregistered on an open science platform. However, it is important to note that the entire research process was monitored by the Medical Ethics Committee of Gansu Provincial People’s Hospital (Approval No. 2020-067, Date: April 2, 2020). According to this committee’s regulations, researchers were required to strictly adhere to the approved protocol for experimental design, data collection, and analysis. Any substantive modifications would have necessitated re-submission for ethical review. This oversight mechanism functionally aligns with the goals of preregistration by ensuring transparency and traceability through third-party auditing, thereby mitigating risks of selective reporting bias. In addition to local institutional review of this study, preregistration is a promising direction for research monitoring and sharing (58), and researchers need to provide standardized detailed research plans and content based on excellent templates (59) to help mitigate positive results bias and publication bias (60, 61).

### 3.3 Conclusion

This article pioneers the investigation of depression correlates in Chinese higher education settings and revealed a negative correlation between participants’ PHQ-9 scores and their dietary regularity, a negative correlation between their PHQ-9 scores and their bedtime, and a negative correlation between their PHQ-9 scores and their overall physical activity level. Simultaneously, we have validated the relationship between smartphone sensor data and depressive symptoms.

Through this research, we have extracted 18 feature sequences that can be used to reflect the relationship between smartphone usage patterns and depressive symptoms in the context of campus life in China, including direct and derived features. We employed classification modeling using five models: Support Vector Machine, K-Nearest Neighbor, Naive Bayes, Decision Tree, and Random Forest, to validate the 18 features, achieving accuracy rates ranging from 73.11% to 88.24%. This research, based on daily behaviors, is of significant importance for the early assessment of depression in Chinese universities, thus contributing to future endeavors in the identification, assessment, and intervention of depression.

## Data Availability

The datasets presented in this study can be found in online repositories. The names of the repository/repositories and accession number(s) can be found in the article/**Supplementary Material**.
