# Supplementary material for: Smartphone sensor-based depression detection in campus environments: a proof-of-concept study with small-sample behavioral analysis
Source: Front Psychiatry. 2025 Aug 7;16:1468334. doi: 10.3389/fpsyt.2025.1468334 (PMC12368363; doi:10.3389/fpsyt.2025.1468334)
Supplement: Supplementary file 1 [file DataSheet1.pdf]

Supplementary Material

1 SUPPLEMENTARY DATA

The dataset analysed in this paper has been uploaded to Zenodo.

DOI: 10.5281/zenodo.12743801

Website: https://doi.org/10.5281/zenodo.12743801

2 SUPPLEMENTARY FIGURES

All Figures are shown in the manuscript.

3 SUPPLEMENTARY TABLES

Table S1. Pearson correlation coefficient between features and degree of depression

Feature	Correlation	Feature	Correlation	Features	Correlation
AACR	-0.481	FL	-0.567	AW	0.631
AA	-0.417	IQR	-0.256	MO	-0.684
GACR	-0.449	Skewness	0.203	NO	-0.421
FG	-0.697	SB	-0.217	AO	-0.667
AG	-0.637	SS	0.203	EO	-0.604
AL	-0.567	AB	0.723	LB	0.672
